# A Mathematical Model of the Thermo-Anemometric Flowmeter

**DOI:** 10.3390/s150922899

**Published:** 2015-09-11

**Authors:** Igor Korobiichuk, Olena Bezvesilna, Andriі Ilchenko, Valentina Shadura, Michał Nowicki, Roman Szewczyk

**Affiliations:** 1 Industrial Research Institute for Automation and Measurements, Jerozolimskie 202, Warsaw 02-486, Poland; E-Mail: kiv_Igor@list.ru; 2 National Technical University of Ukraine “Kyiv Polytechnic Institute”, 37, Avenue Peremogy, Kyiv 03056, Ukraine; E-Mail: bezvesilna@mail.ru; 3 Zhytomyr State Technological University, Chernyakhovskogo str., 103, Zhуtomуr 10005, Ukraine; E-Mails: avi_7@rambler.ru (A.I.); valshad@mail.ru (V.S.); 4 Institute of Metrology and Biomedical Engineering, Warsaw University of Technology, Boboli 8, Warsaw 02-525, Poland; E-Mail: r.szewczyk@mchtr.pw.edu.pl

**Keywords:** thermo-anemometer, flowmeter, TAF, modeling, biofuels, flow, heat transfer, thermal conductivity

## Abstract

A thermo-anemometric flowmeter design and the principles of its work are presented in the article. A mathematical model of the temperature field in a stream of biofuel is proposed. This model allows one to determine the fuel consumption with high accuracy. Numerical modeling of the heater heat balance in the fuel flow of a thermo-anemometric flowmeter is conducted and the results are analyzed. Methods for increasing the measurement speed and accuracy of a thermo-anemometric flowmeter are proposed.

## 1. Introduction

The necessity for fuel consumption control is becoming more and more important in automobile transport design. Flowmeters of various types are aimed to solve this problem and to avoid fuel misuse. Flowmeters are not only used for automobile and freight transport, but also for farm machinery (forklifts, harvesters, tractors and others), for special construction equipment, river and sea transport, buses, *etc*.

There are a lot of fuel flowmeters available. Their classification takes into account parameters such as fuel type, presence and type of output (analog, impulse, and so on), data transmission system type, indicator availability, *etc*. The list of flowmeters in use includes: Coriolis, ultrasound, turbine, thermal, screw, piston, float, and magnetic induction devices. Every flowmeter, among those mentioned above, has its own particularities and drawbacks.

Currently, the thermo-anemometric flowmeter (TAF) is considered one of the best devices for measuring the consumption of biofuels [1]. Its main principle is to heat the fuel flowing to the engine, and to measure the distribution of the temperature field created by the heater in this flow. The changes of temperature field with engine fuel flow are determined by the definite functional dependence on fuel consumption value. That is why it is possible to determine fuel consumption with high accuracy by measuring the temperature field along the engine fuel flow axis. This makes the problem of thermo-anemometric flowmeter mathematical model development of current interest.

The existing thermo-anemometric flowmeters use one or two thermocouples to measure the temperature distribution. These thermocouples are placed directly at the heater, or on its sides at some distance. The mathematical model of existing flowmeters focuses on the flowmeter heat balance equation, or on the determination of the temperature difference between two fixed points.

The new flowmeter design uses groups of thermocouples to measure the engine fuel consumption with higher accuracy. Such a solution provides the determination of temperature value at the fixed set of points within the engine fuel flow, and subsequent computer algorithmic equation processing compensates for a number of measurement errors. Thus, it is necessary to develop a new mathematical model for this flowmeter. This will allow for measurement of the detailed distribution of the temperature field at all points of the fuel flow where the thermocouples are installed. They should be placed in locations where the medium flow rate is the highest, which will result in an increased heat transfer coefficient.

The aim of this paper is to develop the mathematical model of thermo-anemometric flowmeter [2] and to conduct the corresponding modeling. To achieve this, it is necessary to analyze the existing mathematical model of the temperature field in a biofuel flow going through the flowmeter; to conduct the computer numerical modeling of the heater heat balance in mobile fuel stream through a TAF; to develop a new mathematical model of the temperature field distribution along the tube; to calculate the improved value of biofuel consumption and to propose methods to improve the accuracy and speed of TAFs.

In the existing literature concerning flowmeter construction and modeling one can find further information about this issue. Xu *et al*. [3] described the physical principle of an induction meter and the basic formulas for its calculation are presented. The criteria for the E grille induction flowmeter are grounded. In [4] the results of an experimental study on an electromagnetic flowmeter are given. The methods for this study are described and a final result processing algorithm is proposed. The article [5] describes a 3D computer model of a turbine flowmeter and the results of the corresponding modeling are shown.

A precise flowmeter consisting of a couple of cyclical internal rotors is considered in article [6]. This flowmeter is widely used in hydraulic control systems. The available flowmeters for measuring fuel consumption, their design and functioning principles are described in [7,8,9]. Modern thermocouples for TAF designs are considered. Their advantages and disadvantages are examined and the basic characteristics are presented.

## 2. Thermo-Anemometric Flowmeter Design and Principle

A thermo-anemometer is a device for measuring fluid flow speed. Its functioning principle is based on the dependence of convective heat transfer of sensor (S) on the flow speed, when the sensor is placed in the flow and heated by an electric current.

**Figure 1:**
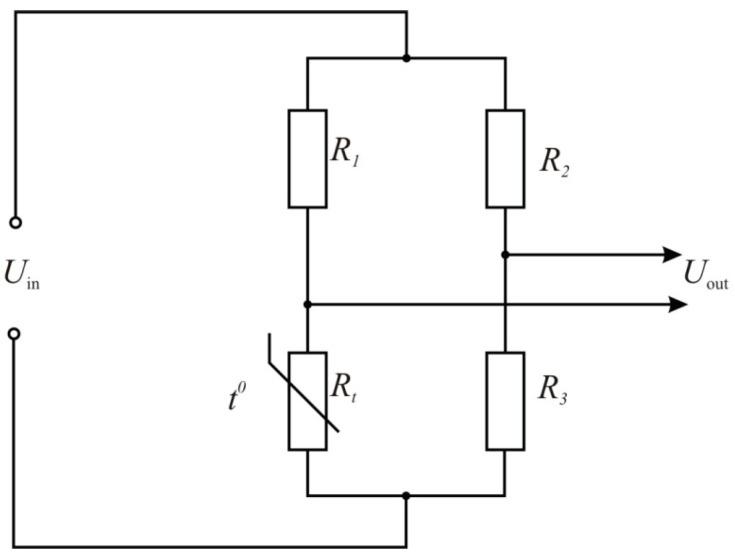
Measuring bridge of a thermo-anemometer (*t*^0^—the measured temperature).

The measuring bridge is the main part of a thermo-anemometer (Figure 1). This bridge has the sensor in one of its arms. The amount of heat, which is transferred by the heated sensor to the fluid flow, depends on the physical characteristics of the moving medium, piping geometry and sensor orientation. The higher the temperature of the sensor is, the higher the sensitivity of the thermo-anemometer is.

Thermo-anemometers are classified according to the features which characterize the heat mode of the converter: the way of sensor heating (direct, indirect, continuous, and impulsive), the type of bridge current (direct, alternating), the type of electric circuit, *etc.* There are direct current and constant temperature thermo-anemometers depending on the converter heat mode.

The bridge of such a generator is powered by a source with high internal resistance. It provides a constant current value at the sensor changing the resistance. Due to the fact that the temperature of the sensor changes with time, the band of recorded frequencies for non-stationary and turbulent flow is limited because of the sensor thermal lag. This causes a decrease of the amplitude of the signal at high frequency ω pulsations of times, where τ is the time constant of the sensor. Thermo-anemometers of the hot wire type use a very fine wire on the order of several micrometers, electrically heated up to some temperature above ambient. Fluid flowing past the wire has a cooling effect on it. As the electrical resistance of most metals is dependent upon the temperature of the metal, a relationship can be obtained between the resistance of the wire and the flow speed.

Several ways of implementing this exist, and hot-wire devices can be further classified as constant current anemometers (CCAs), constant voltage anemometers (CVAs) and constant-temperature anemometers (CTAs). The voltage output from these anemometers is thus the result of some circuit response within the device trying to maintain the specific variable (current, voltage or temperature) constant, following Ohm's law.

Additionally, pulse-width modulation (PWM) anemometers are also used, wherein the velocity is inferred by the time length of a repeating pulse of current that brings the wire up to a specified resistance, and then stops until a threshold “floor” is reached, at which time the pulse is sent again.

**Figure 2:**
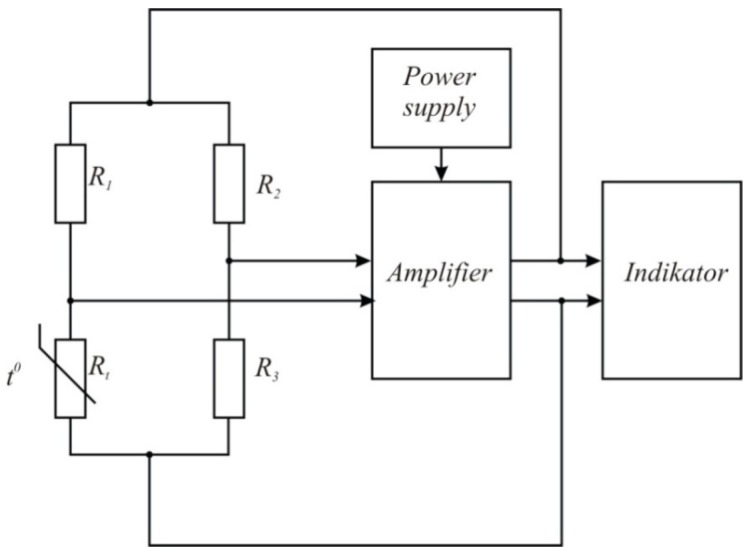
Thermo-anemometer of constant temperature type block diagram (*t*^0^—the measured temperature).

Constant temperature thermo-anemometers (Figure 2) have recently become popular. The main elements of such thermo-anemometers are a measuring bridge with a sensor in one of its arms and a feedback amplifier. The direct current amplifier has to have a high gain (not less than 8000…10,000) and uniform frequency response within the 0–30 kHz band. The reliable functioning of the high frequency amplifier is due to the frequency-dependent feedback. The feedback measurement allows for a wide range of frequency settings.

Despite a number of advantages (low inertia, high sensitivity, high accuracy, reliability) the constant temperature thermo-anemometer has a significant drawback. The indications of this device are dependent on the environmental temperature. This necessitates measuring the temperature in the fuel flow channel, and makes the device diagram more complex. Moreover, the impact of one sensor on another increases the number of measurement errors.

It is important to mention that the grading of thermistors, which are used in the thermo-anemometers as sensors, can be a time-consuming process. That is why it is performed within the limited temperature range in contrast to the real range of working temperatures, which is much wider. Therefore, this may cause the significant increase of measurement errors for the cases when the temperature of fluid or gas is outside of the construction-phase sensor grading range.

Based on the sensor design there are following types of thermo-anemometers:

- Hot Wire Sensor,
- Hot Film Air Flow Sensor.

Hot-wire anemometers, while extremely delicate, have extremely high frequency-response and fine spatial resolution compared to other measurement methods, and as such are almost universally employed for the detailed study of turbulent flows, or any flow in which rapid velocity fluctuations are of interest.

**Figure 3:**
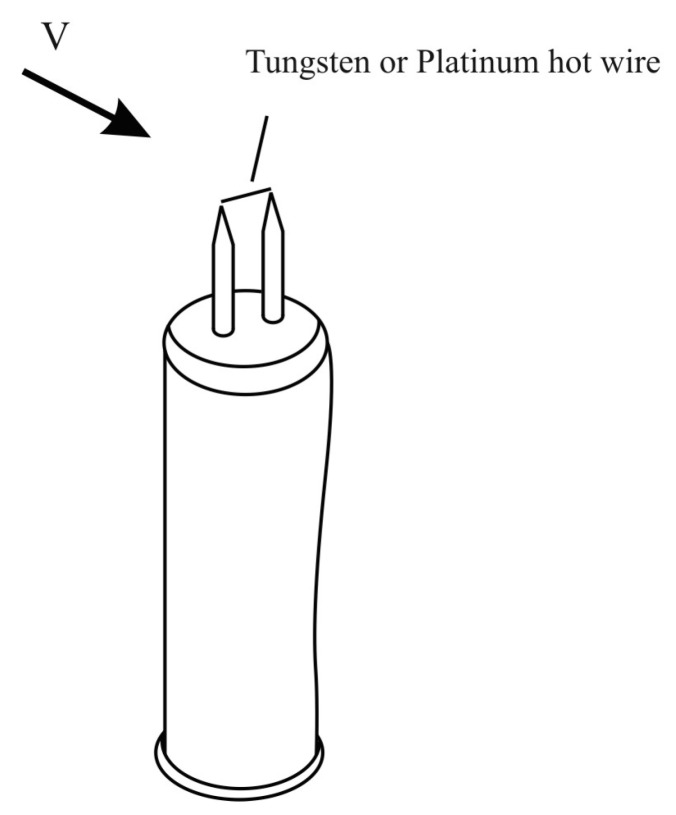
Typical thermo-anemometer diagram [10].

The sensor element in the form of heated platinum filament is the basis of a hot wire sensor. Its main function is to support the constant temperature of the platinum filament by means of electric current heating. Typically, the anemometer wire is made of platinum or tungsten and is 4~10 µm in diameter and 1 mm in length (Figure 3). Typical commercially available hot-wire anemometers have a flat frequency response (<3 dB) up to 17 kHz at the average velocity of 9.1 m/s, 30 kHz at 30.5 m/s, or 50 kHz at 91 m/s.

**Figure 4:**
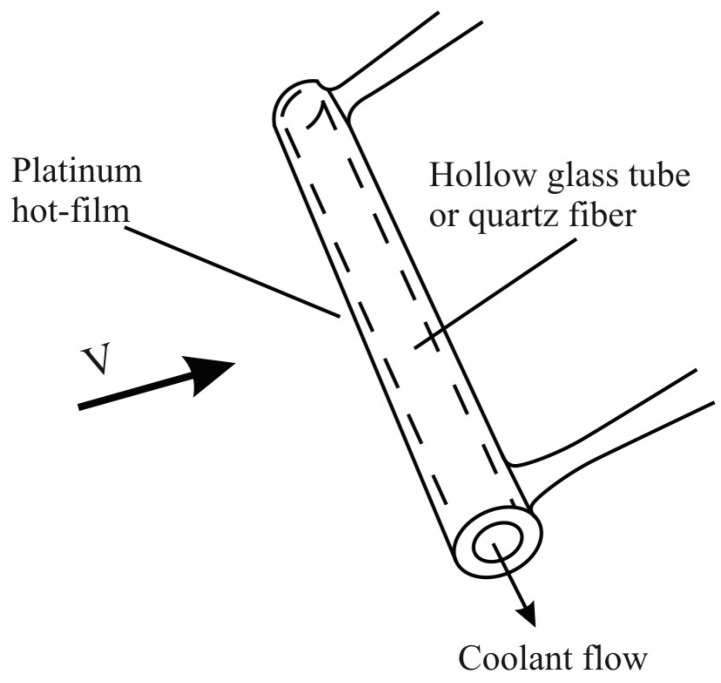
Tube thermo-anemometer diagram [10].

Due to the tiny size of the wire, it is fragile and thus suitable only for clean gas flows. In liquid flow or rugged gas flow, a platinum hot-film coated on a 25~150 mm diameter quartz fiber or hollow glass tube can be used instead, as shown in the schematic below (Figure 4) [10].

**Figure 5:**
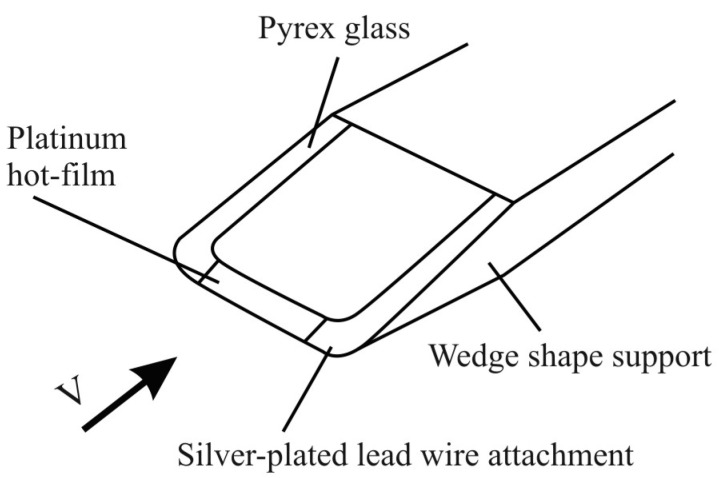
Wedge thin-film thermo-anemometer diagram [10].

Another alternative is a Pyrex glass wedge coated with a thin platinum hot-film at the edge tip, as shown schematically below (Figure 5). The sensor is cooled by the flow of the fluid in which the sensor is positioned. The voltage transformer transforms the electric current changes of the heated element into an output voltage. There is the non-linear dependence of the output voltage on the fluid flow mass. The engine control unit controls this process. Additionally, there is the self-cleaning mode provided for the hot sensor, which starts a brief heating of platinum filament to a temperature of 1000 °C. This prevents sensor pollution.

It is important to mention that the thickness of the platinum filament decreases during the thermo-anemometer operation and so, the precision of measurements is decreased correspondingly.

An industrial version of the fine-wire anemometer is the thermal flowmeter, which follows the same concept, but uses two pins or stings to monitor the variation in temperature. The stings contain fine wires, but encasing the wires makes them much more durable and capable of accurately measuring air, gas, and emissions flows in pipes, ducts, and stacks. Industrial applications often contain dirt that will damage the classical hot-wire anemometer.

The hot film fluid flow sensor does not have such a drawback. That is why it has replaced the hot wire sensor. The hot film sensor principle of operation is the same as the hot wire sensor. The main difference is in the sensor construction. A silicon crystal is the sensing element of a hot film sensor. It is covered with thin platinum or nickel layers, which are the resistors. These resistors are the heating resistor, two thermo-resistors, and the fluid temperature sensor resistor.

The sensor is placed in the specific fluid channel which leads the fluid to the engine. The speed of the flow prevents most of the pollution of the sensor. The channel design allows for the determination of the mass of either forward or reverse (reflected from the closed valves) flows, and this increases the measurement accuracy.

The heating resistor supports a certain temperature of the sensor. The temperature difference at the thermo-resistors helps determine the mass of intake fluid and the direction of the fluid flow. The direct current voltage is the output analog signal of the sensor.

Some of the mass fluid flow sensors designs generate a digital signal instead of an analog one. This is preferable for control systems, because it does not depend on the mode of the device operation and the electrical circuit characteristics. The signals of the hot film sensor are used by the control unit to determine the following parameters:

For petrol engines they are the injection time, the amount of injected fuel, the ignition timing, the order of system operation for petrol vapor;
For diesel engines they are the injection time, and the order of the exhaust gases recirculation system.

The thermo-anemometric method is currently used to measure fuel consumption. This method is based on the dependence of the sensor convective heat transfer on the flow speed, when the sensor is placed into the flow and heated by the electric current. The electronic fuel flow meter does not have rotating elements and is able to operate in the polluted fuel medium in contrast to other pipeline flow meters.

Its sensor is of a differential type and this means that it measures the value that is proportional to PW at one point, so it is important to know the dependence of the sensor readings on its location.

The thermo-anemometers of fuel and air media have many advantages compared to other types. They allow for measuring the amount of substances (kg/s). This is a parameter which is not dependent on pressure, temperature, *etc.* The sensors are constructed on the basis of stable metal film resistors, and this provides better metrological characteristics to the devices mentioned above.

Thermo-anemometrical flowmeters aim to measure any amount of fuel and air media immediately. They can be also used as gas meters, for measuring the air flow of the industrial ventilation and so on. These flowmeters possess great advantages: they are compact and able to resist temperatures ranging from −30 °C to +100 °C.

The sensors of the thermo-anemometer are a couple of thermo-dependent resistors which are placed in the fluid pipeline. The thermo-anemometer temperature regulator sends an electric current into one of the sensor resistors. This current heats it to the temperature *Т_h_* which exceeds the temperature T_e_ in the pipeline. The value of the formed current is automatically set to make the overheating *Т_h_ – T_e_* constant at the first approximation independently from T_e_ and the velocity of gas flow *V* in the pipeline. Here, the heat capacity *W_h_* between the resistance and gas is expressed by

(1) $$W_{h}=(T_{h}-T_{e})Q(\rho V)$$

where ρ is the density of fluid in the pipeline, *V* is the fluid flow velocity, *Q* is the coefficient that is determined by the product of *ρV.* and it is specified for the system’s geometrical parameters and the chemical composition of the fluid in the pipeline. The electric power at the resistor is equal to:

(2) $$W_{i}=r_{I}I^{2}$$

where $r_{I}$ is the heat resistor resistance (in ohms), *I*—the current coming through the resistance (in amperes).

The equation $W_{h}=W_{i}$ is true at the constant temperature *Т_h_*, thus, there is the ratio:

(3) $$I=\sqrt{\left[(T_{h}-T_{e})Q(\rho V)/r_{I}\right]}$$

The signal which is formed at the thermo-anemometer input is proportional to the *I*, so, it is the function of *ρV* value that can be considered as the transfer function of the thermo-anemometer. The functional converter is used to implement the functional dependence, which is the inverse (with the precision to the scale coefficient) to the transfer function of the thermo-anemometer. It provides the appearance of the signal at the input of the functional converter which is proportional to the *ρV* value.

The integrator produces the time integration of the value representing the result in the form of a number that is proportional to the mass of fluid which has come through the section of the pipeline at the thermo-anemometer installation point. The proportion coefficient is determined by the ratio of the cross section of the pipeline to the constant of integrator time multiplied by the scale coefficient *k*.

The thermo-dependent resistors are nickel-based. The temperature dependence of the resistances *r_1_* and *r_2_* can be represented by the linear function in the temperature interval [*Т_h_*…*T_e_*], assuming that:

(4) $$\begin{array}{l} r_{1}(T)=w_{1}(T_{h}-T_{0})\\ r_{2}(T)=w_{2}(T_{e}-T_{0}) \end{array}$$

where *w_1_* and *w_2_* are the coefficients, determined by the resistor design.

**Figure 6:**
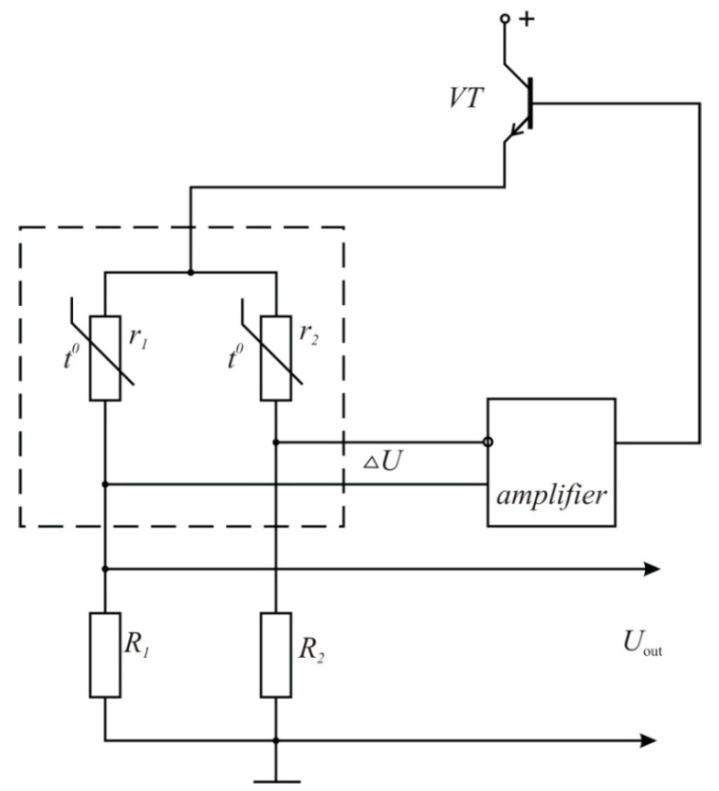
The thermo-anemometer electric circuit.

**Figure 7:**
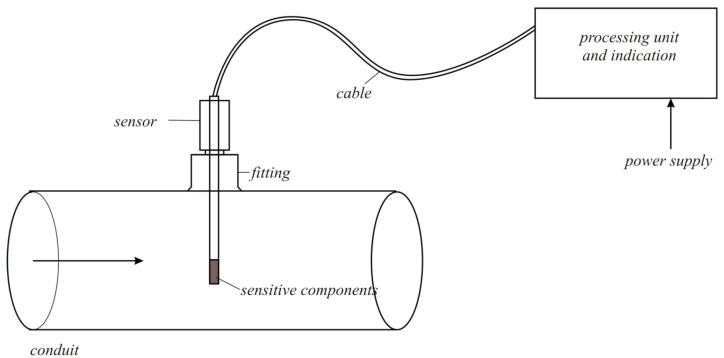
The thermo-anemometric flowmeter schematic diagram.

The sensor design makes the condition *w_1_ << w_2_* true, which is equivalent to *r_1_ << r_2_*. As a result, the current coming through *r*_2_, as well as the power dissipated at this resistor at the same voltage, are small as compared to the same parameters of *r*_1_. It is important to remember that the heat sink from the resistor *r*_2_ coming through the metal construction is more efficient than the heat sink from the resistor *r*_1_, so we can conclude that the resistor temperature does not depend on the operating voltage *U*, but it coincides with the environmental temperature (fluid temperature *Т_h_*). The presence of this resistor in the thermo-anemometer circuit allows for implementation of the temperature stability of the transfer factor.

Figure 6 shows the thermo-anemometer electric circuit. Figure 7 shows the schematic diagram of the thermo-anemometric flowmeter, the sensor, and the processing and display unit.

## 3. The Mathematical Model

The proposed mathematical model of temperature field in biofuel stream coming through thermo-anemometric flowmeter shows the following peculiarities:

- the heater operates in continuous power mode,
- thermocouples are placed along the axis of the biofuel stream, and the temperature distribution is determined along one special coordinate,
- laminar and turbulent modes of the biofuel stream are considered,
- due to the fact that the range of initial temperatures is rather wide in TAF application, the TAF has been improved taking into account temperature changes.

While calculating heat flow between heater surface and biofuels medium, we represent the density of heat flow in the form of a law [2,11]:

(5) q=αΔT

where *ΔТ* is temperature difference between surface and environment; *α*—is convective heat transfer coefficient.

The equation for a biofuel heat stream in a stationary medium, taking into account thermal conductivity, has the form:

(6) $$Q=-\lambda A\frac{dT}{dR}=-\lambda 4\pi R^{2}\left(-\frac{R_{H}}{R^{2}}\right)\left(T_{0}-T_{H}\right)=4\pi \lambda R_{H}\left(T_{0}-T_{H}\right)$$

where *Т_H_*—is temperature on heater surface; *Т_0_*—is biofuels initial temperature.

The heat flux radius *R_H_* and surface area *S_H_* can be calculated using the Equation (5):

(7) $$Q=qS_{H}=4\pi R_{H}^{2}\alpha \left(T_{0}-T_{H}\right)$$

we get $\alpha =\lambda /R_{H}$ from Equations (5) and (6).

The expression for calculation of the temperature of heater at biofuels movement speed ν = const gets the following form:

(8) $$T_{H}=T_{0}+\frac{P}{\pi l_{H}K_{1}}{\left(\frac{\pi \nu d_{тр}}{4W}\right)}^{K_{2}}{\left(\mu C\right)}^{-K_{3}}{\left(\lambda \right)}^{1-K_{3}}$$

here *Р—*the heater power; *μ—*the biofuels dynamic viscosity coefficient; *С*, *λ*—the biofuel heat capacity and viscosity; *d_тр_*—the TAF tube diameter; *K_1_ = K_2_* = 0.5; *l_H_*—the distance to the heater; *W—*the biofuel volumetric rate:

(9) $$W=K_{4}K_{5}{\left(T_{0}-T_{H}\right)}^{-1/K_{2}}$$

*K_4_*—the coefficient taking into account TAF constructive parameters:

(10) $$K_{4}=\frac{\pi d_{тр}}{4}{\left(\frac{P}{\pi l_{H}}\right)}^{1/K_{2}}$$

(11) $$K_{5}=\nu {\left(\mu С\right)}^{K_{3}/K_{2}}{\left(\lambda \right)}^{\left(K_{3}-1\right)/K_{2}}K_{1}^{-1/K_{2}}$$

*K_5_*—the coefficient taking into account physical and chemical properties of biofuels and the mode of flow through the flowmeter.

## 4. Heater Heat Balance Modeling in a Mobile Fuel Flow

**Figure 8:**
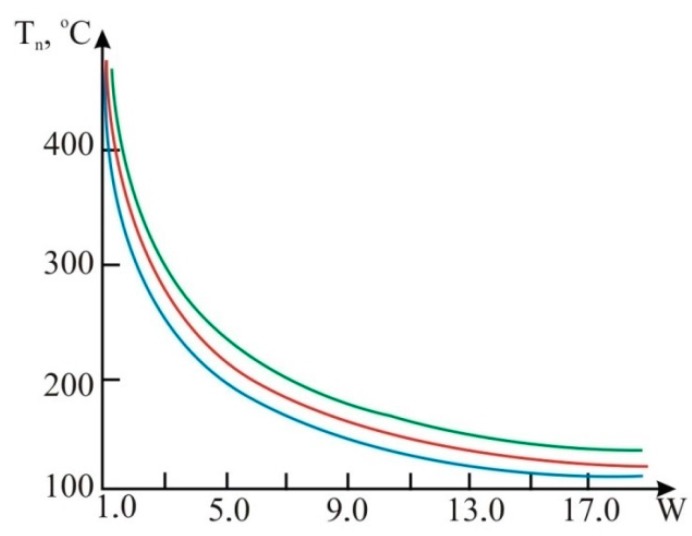
The graphs of temperature dependence on fuel consumption: 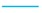 gasoline; 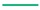 diesel fuel; 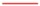 biofuels Т_n_, °C.

**Figure 9:**
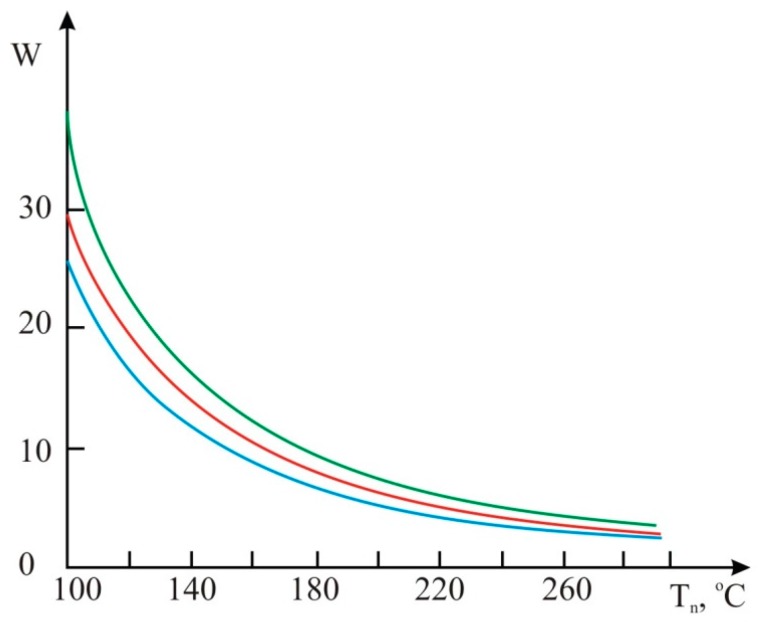
The graphs of fuel consumption dependence on heater temperature: 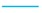 gasoline; 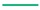 diesel fuel; 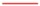 biofuels.

Heater heat balance in mobile fuel flow through a TAF is calculated using Equations (7) and (8). The physical and mathematical parameters of the fuel are given in Table 1 and design parameters are given in Table 2. The results of the TAF heater heat balance in accordance with the Equations (7) and (8) are shown in Figure 8 and Figure 9. We can see that it is necessary to measure engine fuel temperature simultaneously at the corresponding points of flow. This will significantly increase TAF accuracy. We get the mathematical Model (15) for the temperature field distribution along tube and the improved fuel consumption value.

**Table 1:** Physical parameters of the fuels.

№	Parameter	Gasoline	Diesel Fuel	Biofuels
1	Density, kg/m^3^	750.0	805.0	865.0
2	Thermal conductivity, kJ/kg·K	2.20	2.10	1.50
4	Thermal diffusivity, m^2^/s	6.67 × 10^−8^	6.51 × 10^−8^	1.32 × 10^−7^
5	Kinetic viscosity coefficient, m^2^/s	4.93 × 10^−7^	2.11 × 10^−6^	4.25 × 10^−6^

**Table 2:** TAF construction parameters.

Parameter	Values for Cases		
	I	II	III
Tube diameter, mm	15	10	5
Tube length, mm	300	300	300
Heater diameter, mm	3	3	1
Heater length, mm	12	12	12
Heater power, W	12	12	12

The new formula for the determination of fuel temperature along the axis of a TAF tube for the movement of fuel and laminar flow of the engine fuel inside tube has the form:

(12) $$Т(х)={Т}_{н}-\left[\frac{\pi х\alpha }{W}-\frac{7}{48}\right]\frac{4P}{\pi d_{тр}\lambda }$$

Measuring the heater surface temperature and biofuel temperature at a distance x from the heater by means of the TAF, we determine the biofuel volumetric flow rate:

(13) $$W=\frac{\pi х\alpha }{\frac{\pi d_{тр}\lambda ({Т}_{н}-T(x))}{4P}+\frac{7}{48}}$$

The final expression for heater temperature calculation under turbulent biofuel flow has the form:

(14) $$T\left(x\right)=T_{H}-Texp\left\{-0.11{\left(\frac{\pi }{4}\right)}^{0.2}\frac{x{\left(v\right)}^{0.2}}{{\left(d_{mp}\right)}^{0.8}{\left(W\right)}^{0.2}}\right\}+T$$

Measuring the heater surface temperature by means of a TAF, the environmental temperature and the temperature of the biofuel at a distance x from the heater, we determine the biofuel volumetric rate:

(15) $$W={\left(\frac{K_{8}K_{9}x}{\ln\left[Т(х)-T\right]-\ln\left[{Т}_{н}-T\right]}\right)}^{5}$$

where *K_8_* = −0.11(π/4)^0.2^, *K_9_* = (ν)^0.2^/(*d_тр_*)^0.8^.

In order to increase the accuracy of measurements in laminar and turbulent modes T(x), it is recommended to apply the procedure of linear approximation at several points of the biofuel flow.

**Figure 10:**
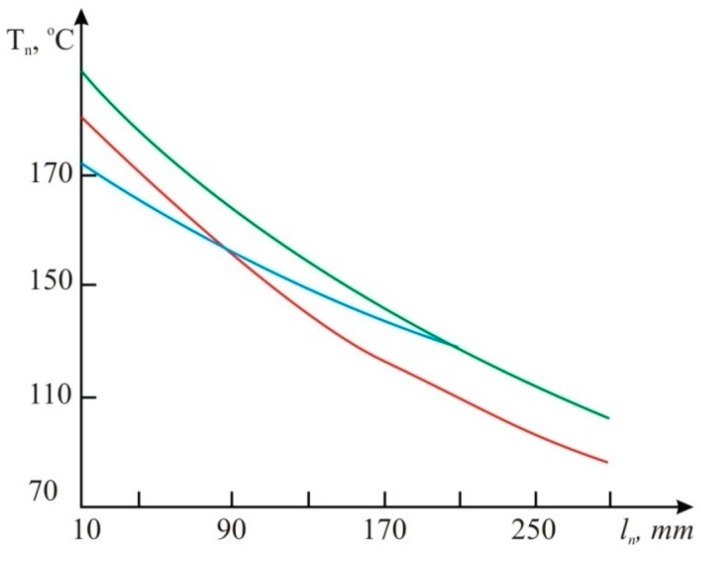
The graphs of dependence *T*_n_ on *l*_н_(*d_тр_=15* mm; *d_н_=3* mm): 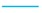 gasoline; 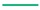 diesel fuel; 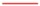 biofuels, the thermocouple error is 5 °С.

Having completed computer modeling of regular volumetric fuel consumption of 5 L/h for the various types of engine fuel (gasoline, diesel fuel and biofuel), and different distances from the heater, we get the results represented in Figure 10. For construction of graphs presented in Figure 10 we used Equation (8), in which the temperature *T_H_* was calculated depending on the distance to the heater *l_h_* for different fuels (Table 1). The best fitness is provided with the ANOVA method for multiple factors (Figure 10).

**Figure 11:**
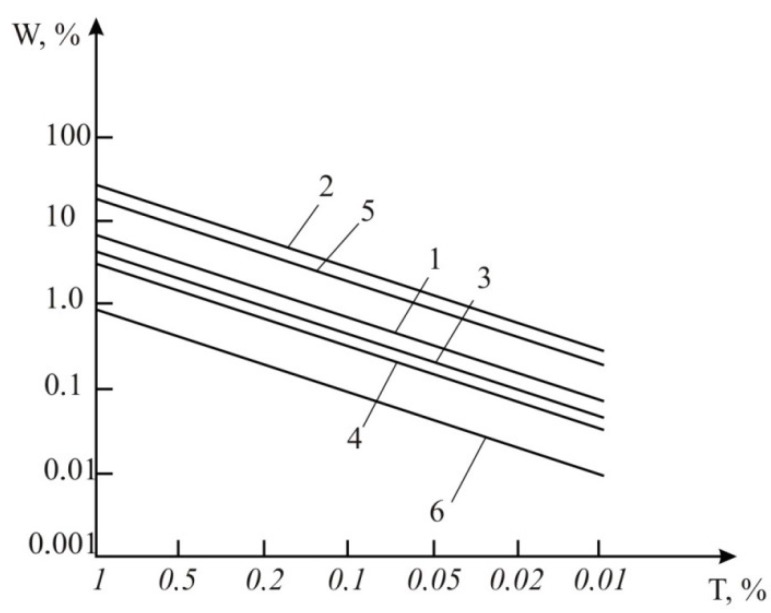
The results of computer modeling of TAF errors using biofuels for variants 1-6.

The obtained results show that the volumetric fuel consumption depends on the thermocouples’ temperature measurement errors within the range of relative errors from 0.01 to 1.0%. This error was calculated using the relative method. These methods are described in [2,12] or result from the use of Equations (8) and (15). TAF error modeling is performed by using an engine fuel with the mathematical and mechanical properties presented in Table 1 for the following cases (Figure 11):

(1) The consumption determination according to Equation (8) based on the results of heater temperature measurements and the engine fuel initial temperature (variant 1).
(2) The consumption determination according to Equation (15) by the results of engine fuel initial temperature measurement and the temperature of engine fuel at two points along the tube axis with the average value for two points (variant 2).
(3) The accuracy increase of variant 2 as a result of algorithmic compensation of random and dynamic errors by means of artificial neural networks [12].
(4) The accuracy improvement of variant 1 by means of approximation the results of thermocouple temperature measurement and error compensation for these measurements. [2].
(5) The accuracy improvement of variant 2 by means of approximation the results of thermocouple temperature measurement and error compensation for these measurements [2].
(6) The accuracy improvement of variant 2 by means of:
  - the algorithmic compensation of random and dynamic errors by using artificial neural networks [12];
  - the approximation of results temperature change caused by thermocouples and error compensation for these measurements [12].

The results of computer modeling of TAF errors using biofuels for variants 1–6 are presented in Figure 11. Graph number 1 was plotted based on the results of experimental studies (measurements of the temperature of the heater and the initial temperature of the fuel) and based on Equation (8). Graph number 2 was plotted by measuring the initial temperature of the fuel and averaging the temperature measured at two points along the tube axis, based on Equation (15). Graph number 3 is plotted on the basis of graph 2 with applied algorithmic compensation of random and dynamic errors using artificial neural networks [11]. Graph number 4 was plotted based on graph 1 using an approximation of the results of thermocouple temperature measurement and compensation of the measurement errors [2]. Graph number 5 was plotted based on graph 2 using the same procedure as in the case of graph 4. Graph number 6 was plotted based on graph number 2 with applied algorithmic compensation of random and dynamic errors using artificial neural networks, and also approximation of results of thermocouple temperature measurement and compensation of these measurements [11]. As we can see in Figure 11, it is advisable to use variant 1 with a minimum number of mathematical calculations. It will provide an accuracy of volumetric fuel consumption W = 3% at a temperature measurement accuracy of the heater of 1%.

## 5. The Results Analysis

The designed mathematical model of the temperature field in a biofuel stream coming through a flowmeter is analyzed. The computer numerical modeling for the heater heat balance in a mobile fuel flow coming through a TAF is performed. It is found that in order to increase the accuracy of the TAF, it is recommended to measure the temperature of the engine fuel simultaneously at the corresponding points of flow. The existing flowmeters use one or two thermocouples to measure thermal field, and their mathematical model is based on the equation of heat balance of a heater which is cooled by a liquid stream or by the determination of the temperature difference at two fixed points. The new highly precise flowmeter uses groups of thermocouples to increase the accuracy of engine fuel consumption measurements. Such a solution provides the determination of the temperature field value at a set of engine flow points and the subsequent algorithmic computer processing of the obtained values compensates for any measurement errors. Thus, after the results are processed, we get the new mathematical model of the temperature field along a tube, and the improved value of biofuel flow as well.

In order to improve the measurement accuracy at laminar and turbulent modes, it is advisable to conduct measurements at several points of the biofuel stream and to apply the procedure of linear approximation. Computer modeling is performed, and it is done at a constant volumetric flow rate of fuel for the different types of engine fuel, and at different distances from the heater. The results obtained show that volumetric flow rate depends on errors of temperature measurements of thermocouples in the range of relative errors from 0.01% to 1.0%.

The results show, that in order to improve the TAF accuracy it is recommended to apply more complex procedures, such as approximation of the measurement results by a least squares method and artificial neural networks. To increase TAF speed it is advisable to install a specialized neuroprocessor in the computer hardware. This will allow for compensation of random and dynamic errors by using artificial neural networks.

## 6. Conclusions

The paper shows the obtained results, which are:

- The existing mathematical model of temperature field in biofuels stream coming through a flowmeter for its components determination is analyzed.
- The modeling of heater heat balance in a mobile fuel flow through a TAF is conducted to identify the basic parameters of the mathematical model, which determine TAF accuracy and speed. The analysis of the results showed the necessity of measuring the engine fuel temperature at the corresponding points of flow in order to improve TAF accuracy.
- A new mathematical model of the temperature field distribution along the tube is developed and an improved biofuel consumption value is calculated.
- New methods of TAF accuracy and speed improvement by using modern neuroprocessors are proposed. The given method allows for compensating the influence of TAF random and dynamic errors.
